# Supplementary material for: Iterative improvement in the automatic modular design of robot swarms
Source: PeerJ Comput Sci. 2020 Dec 7;6:e322. doi: 10.7717/peerj-cs.322 (PMC7924708; doi:10.7717/peerj-cs.322)
Supplement: Supplemental Information 3 [file peerj-cs-06-322-s003.zip › argos3/doc/api/standalone/a00505_source.html]

ARGoS: plugins/robots/generic/control\_interface/ci\_pan\_tilt\_camera\_actuator.h Source File


- Main Page
- Related Pages
- Namespaces
- Classes
- Files

- File List
- File Members

# plugins/robots/generic/control\_interface/ci\_pan\_tilt\_camera\_actuator.h

Go to the documentation of this file.

```
00001 
00007 #ifndef CI_PAN_TILT_CAMERA_ACTUATOR_H
00008 #define CI_PAN_TILT_CAMERA_ACTUATOR_H
00009 
00010 /* To avoid dependency problems when including */
00011 namespace argos {
00012    class CCI_PanTiltCameraActuator;
00013 }
00014 
00015 #include <argos3/core/control_interface/ci_actuator.h>
00016 #include <argos3/core/utility/math/angles.h>
00017 
00018 namespace argos {
00019 
00020    class CCI_PanTiltCameraActuator : public CCI_Actuator {
00021 
00022    public:
00023 
00024       virtual ~CCI_PanTiltCameraActuator() {}
00025 
00032       virtual void SetPan(const CRadians& c_pan) = 0;
00033 
00041       virtual void SetTilt(const CRadians& c_tilt) = 0;
00042 
00043 #ifdef ARGOS_WITH_LUA
00044       virtual void CreateLuaState(lua_State* pt_lua_state);
00045 #endif
00046 
00047    };
00048 
00049 }
00050 
00051 #endif
```

---

Generated on 10 Jul 2018 for ARGoS by 
 1.6.1
